# Low circulating PCSK9 levels in *LPL* homozygous children with chylomicronemia syndrome in a syrian refugee family in Lebanon

**DOI:** 10.3389/fgene.2022.961028

**Published:** 2022-08-19

**Authors:** Carine Ayoub, Yara Azar, Dina Maddah, Youmna Ghaleb, Sandy Elbitar, Yara Abou-Khalil, Selim Jambart, Mathilde Varret, Catherine Boileau, Petra El Khoury, Marianne Abifadel

**Affiliations:** ^1^ Laboratory of Biochemistry and Molecular Therapeutics (LBTM), Faculty of Pharmacy, Pôle Technologie- Santé, Saint Joseph University of Beirut, Beirut, Lebanon; ^2^ Laboratory for Vascular Translational Science (LVTS), INSERM, Paris Cité University and Sorbonne Paris Nord University, Paris, France; ^3^ Faculty of Medicine, Saint Joseph University of Beirut, Beirut, Lebanon; ^4^ Genetic Department, AP-HP, Hôpital Bichat, Paris, France

**Keywords:** chylomicronemia syndrome, type 1 hyperlipoproteinemia, LPL gene, triglycerides, Lebanon, Syria, PCSK9 levels

## Abstract

Familial chylomicronemia syndrome is a rare autosomal recessive disorder of lipoprotein metabolism characterized by the presence of chylomicrons in fasting plasma and an important increase in plasma triglycerides (TG) levels that can exceed 22.58 mmol/l. The disease is associated with recurrent episodes of abdominal pain and pancreatitis, eruptive cutaneous xanthomatosis, lipemia retinalis, and hepatosplenomegaly. A consanguineous Syrian family who migrated to Lebanon was referred to our laboratory after perceiving familial chylomicronemia syndrome in two children. The *LPL* and *PCSK9* genes were sequenced and plasma PCSK9 levels were measured. Sanger sequencing of the *LPL* gene revealed the presence of the p.(Val227Phe) pathogenic variant in exon 5 at the homozygous state in the two affected children, and at the heterozygous state in the other recruited family members. Interestingly, PCSK9 levels in homozygous carriers of the p.(Val227Phe) were ≈50% lower than those in heterozygous carriers of the variant (*p*-value = 0.13) and ranged between the 5th and the 7.5th percentile of PCSK9 levels in a sample of Lebanese children of approximately the same age group. Moreover, this is the first reported case of individuals carrying simultaneously an *LPL* pathogenic variant and *PCSK9* variants, the L10 and L11 leucine insertion, which can lower and raise low-density lipoprotein cholesterol (LDL-C) levels respectively. TG levels fluctuated concomitantly between the two children, were especially high following the migration from a country to another, and were reduced under a low-fat diet. This case is crucial to raise public awareness on the risks of consanguineous marriages to decrease the emergence of inherited autosomal recessive diseases. It also highlights the importance of the early diagnosis and management of these diseases to prevent serious complications, such as recurrent pancreatitis in the case of familial hyperchylomicronemia.

## Introduction

Genetic rare diseases usually require special care that could be challenging and hardly achievable, especially in war crises or migration situations. This is the case of type I hyperlipoproteinemia (T1HLP).

T1HLP (OMIM 238600), also known as familial hyperchylomicronemia, familial lipoprotein lipase deficiency (Pingitore et al., 2016), or familial chylomicronemia syndrome (FCS) (Caddeo et al., 2018), is a rare autosomal recessive disorder of lipoprotein metabolism characterized by the presence of chylomicrons in fasting plasma and an important increase in plasma triglycerides (TG) levels that can exceed 2,000 mg/dl (22.58 mmol/l) (Burnett et al., 1993). This condition is associated with recurrent episodes of abdominal pain and pancreatitis, eruptive cutaneous xanthomatosis, lipemia retinalis, and hepatosplenomegaly (Brunzell and Deeb, 2019). Recurrent episodes of pancreatitis in these patients can affect the functions of the pancreas and can, in severe cases, lead to a multi-organ failure and an increase in morbidity and mortality (Regmi and Rehman, 2021). The prevalence of the disease in the general population is estimated to be one to two per million (Abifadel et al., 2004; Rodrigues et al., 2016), however, it is higher in some isolated ethnic groups (i.e., French Canadians, Afrikaner) (Gagné et al., 1989; Henderson et al., 1992; Foubert et al., 1996; Pingitore et al., 2016), and in populations with a high incidence of consanguinity. T1HLP is caused in most cases by loss-of-function variants in the lipoprotein lipase (*LPL*) gene (Langlois et al., 1989; Caddeo et al., 2018). The latter encodes a secreted protein that plays a crucial role in lipid metabolism and homeostasis through the hydrolysis of TG transported by TG-rich lipoproteins [very low-density lipoproteins (VLDL) and chylomicrons] to decrease plasma TG and generate free fatty acids that are either stored in the adipose tissue or oxidized by the muscles (Wion et al., 1987; Pingitore et al., 2016; Rodrigues et al., 2016). In rarer cases, T1HLP is caused by variants in the apolipoprotein C2 (*APOC2*) (Catapano and Capurso, 1986; Cox et al., 1988; Zanelli et al., 1994) and apolipoprotein A5 (*APOA5*) (Pennacchio and Rubin, 2003; Priore Oliva et al., 2005) genes encoding respectively ApoC2 and ApoA5 which are LPL activators, the glycosylphosphatidylinositol-anchored high-density lipoprotein-binding protein 1 (*GPIHBP1*) gene which encodes a protein that plays a role in the transport and binding of LPL to the endothelial cell wall and its entry into the capillaries (Beigneux et al., 2009; Davies et al., 2010; Song et al., 2022), and in the lipase maturation factor 1 (*LMF1*) gene encoding an endoplasmic reticulum membrane protein, that plays a role in the posttranslational folding, assembly and stabilization of active homodimerized LPL (Péterfy et al., 2007; Péterfy, 2012).

An early diagnosis of the disease is important in order to instore as early as possible a very low-fat diet consisting of reducing the dietary fat to ≤20 g/day or 15% of the total daily energy intake to prevent abdominal pain and recurrent pancreatitis. The goal is to maintain plasma TG levels below 1,000 mg/dl (11.29 mmol/l). It is noted that recurrent abdominal pain is prevented when TG levels are maintained below 2,000 mg/dl (22.58 mmol/l) (Burnett et al., 1993). However, the compliance to the diet is usually poor (Caddeo et al., 2018), and its control is difficult, especially among migrants or low-income populations.

Interestingly, patients with chylomicronemia syndrome generally present low low-density lipoprotein cholesterol (LDL-C) and high-density lipoprotein cholesterol (HDL-C) levels besides high TG levels (Fojo and Brewer, 1992; Hegele, 2013; Blom et al., 2018; O’Dea et al., 2019). More recently, *PCSK9* has been identified as a major protagonist in lipid metabolism and familial hypercholesterolemia (FH) (Abifadel et al., 2003). Many studies have reported a potential correlation between plasma PCSK9 levels and LDL-C, but also TG, VLDL-C, and intermediate-density lipoprotein cholesterol (IDL-C). Investigations linking these actors of the lipid pathway are being conducted (Lakoski et al., 2009; Druce et al., 2015; Norata et al., 2016; Baragetti et al., 2018; Warden et al., 2020).

In this article, we report the case of a consanguineous family of Syrian refugees in Lebanon with two children suffering from familial hyperchylomicronemia. To our knowledge, this is the first study to measure circulating PCSK9 levels in familial hyperchylomicronemia and to identify individuals carrying simultaneously variants in the *LPL* and the *PCSK9* genes.

## Materials and methods

### Study participants

A Syrian family who migrated to Lebanon was referred to our laboratory upon perceiving familial hyperchylomicronemia in two children. The parents and four of their children were recruited. We collected the clinical history and anthropometric data for the recruited members. The parents signed the informed consent to participate with their children in our study. The study was conducted according to the guidelines of the Declaration of Helsinki and approved by the Ethics Committee of Hôtel Dieu de France Hospital and the Saint Joseph University of Beirut.

### Laboratory and biochemical tests

Blood samples were obtained after overnight fasting, plasma, and serum were prepared and stocked at −80°C. Lipid measurements were determined on a COBAS INTEGRA^®^ analyzer (Roche Diagnostics, Basel, Switzerland). Non-HDL-C was calculated by subtracting HDL-C from total cholesterol (TC).

### DNA analysis and variant detection

Genomic DNAs of all the participants were extracted from peripheral blood leukocytes using the illustra^™^ blood genomicPrep Mini Spin Kit according to the manufacturer’s instructions. The exons and the flanking exon-intron boundaries of the *LPL* and *PCSK9* genes were amplified by polymerase chain reaction (PCR) and sequenced using the Sanger method. PCR conditions and primers’ sequences are available upon request. For DNA sequence assembly and variant detection, the CodonCode Aligner^®^ Software was used.

### In silico analysis of the variant

The Genome Aggregation Database (gnomAD; http://gnomad.broadinstitute.org/) was used for frequency determination of the variant. The Polymorphism Phenotyping version 2 (PolyPhen-2; http://genetics. bwh.harvard.edu/pph2/), Protein Variation Effect Analyzer (PROVEAN; http://provean.jcvi.org/index.php), Mutation Taster (http://www.mutationtaster.org/), and the Combined Annotation Dependent Depletion score (CADD score; https://cadd.gs.washington.edu/snv) were used to predict the pathogenicity of the variant.

### PCSK9 measurements

We measured PCSK9 levels in the plasma of the recruited members using a commercial ELISA kit (Human Proprotein Convertase 9/PCSK9 Duoset catalog no. DY3888; R&D Systems, Minneapolis, MN, United States) and the Bio-Plex Pro assay technology (Luminex Corporation, Austin, TX, United States) as previously described (El Khoury et al., 2018).

### Statistical analysis

The variables were analyzed using the GraphPad Prism version 9. Results for quantitative variables were expressed as median with its interquartile ranges (first quartile and third quartile). Spearman correlation was performed to measure the strength and direction of the linear relationship between PCSK9 and other quantitative variables. The Mann–Whitney U test was used to compare PCSK9 values between homozygous and heterozygous carriers of the p.(Val227Phe) variant in the *LPL* gene.

## Results

### Clinical characteristics and biochemical analysis

**FIGURE 1:**
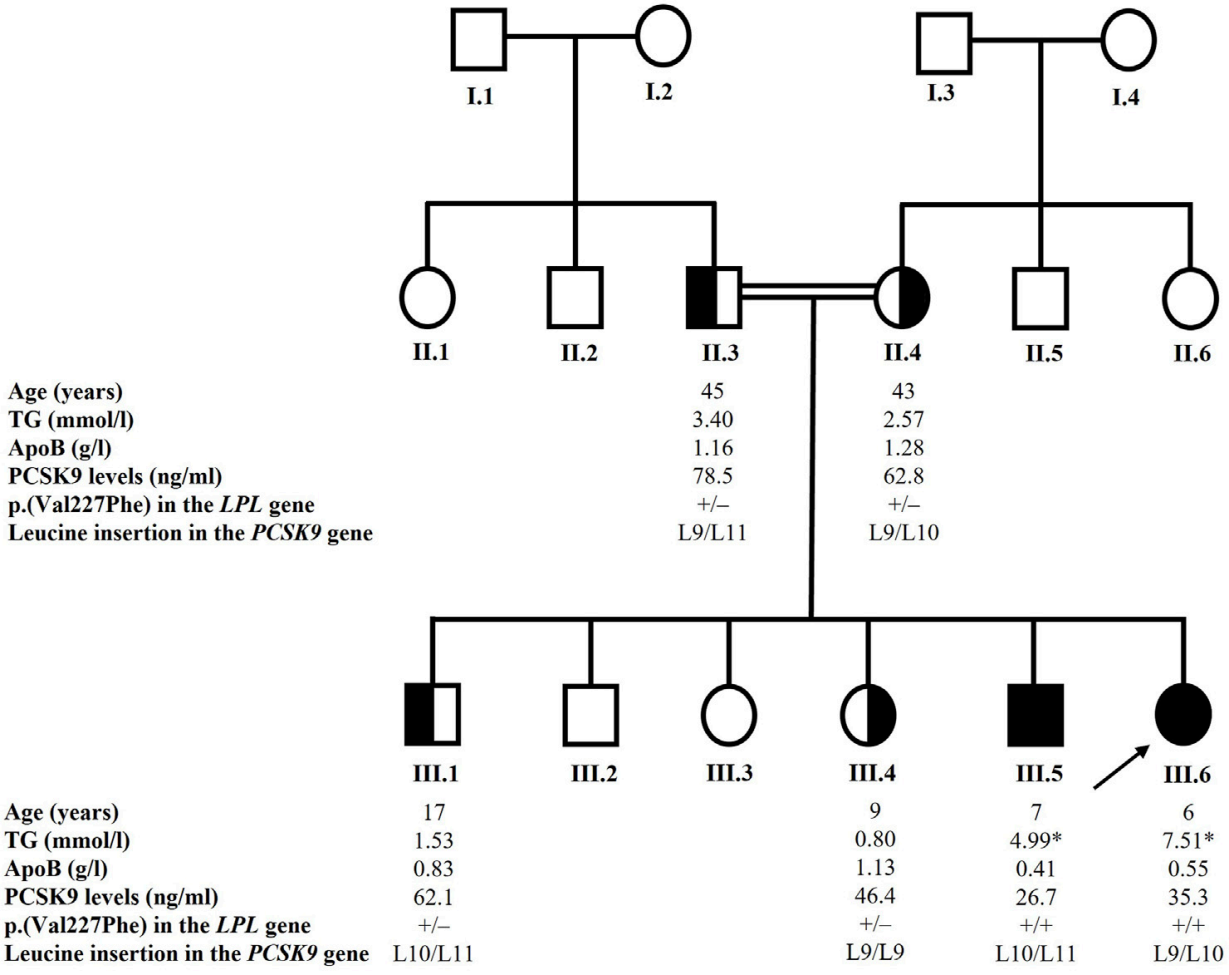
Pedigree of the family. The arrow indicates the proband. Blackened symbols indicate affected homozygous carriers of the p.(Val227Phe) variant in the *LPL* gene. Half-blackened symbols indicate heterozygous carriers of the p.(Val227Phe) variant. Only individuals with available and reported data were included in the study. The * sign indicates TG value while the patient was under a very strict low-fat diet. The +/+ sign indicates that the individual is homozygous for the p.(Val227Phe) variant in the *LPL* gene and the +/– sign indicates that the individual is heterozygous for the variant. The L9 designates the normal allele in exon 1 of the *PCSK9* gene, the L10 designates the p.Leu21dup or p.L15_L16insL and the L11 variant designates the p.Leu21tri or p.L15_L16ins2L. Age at recruitment is given in years. TG levels are given in mmol/l, ApoB levels are given in g/l and PCSK9 levels are given in ng/ml. ApoB: apolipoprotein B; LPL: lipoprotein lipase; PCSK9: proprotein convertase subtilisin/kexin type 9; TG: triglycerides.

In the recruited family, the parents were first-degree cousins and two of their six children suffered from familial hyperchylomicronemia confirmed by lipoprotein electrophoresis and later by genetic sequencing (Figure 1).

The proband (III.6) was a girl aged 6 years at the time of recruitment. She presented high TG levels (7.51 mmol/l) and low LDL-C and HDL-C levels (0.38 and 0.33 mmol/l respectively). At the age of 9 years, she was underweight with a BMI of 16.5 kg/m^2^. She suffered from recurrent abdominal pain, vomiting, and diarrhea almost every week, and the echography showed that she presented splenomegaly. Infectious gastrointestinal causes were ruled out. She suffered as well from chronic anemia. Thalassemia was excluded by the normal results of hemoglobin electrophoresis. Mediterranean fever was suspected and the *MEFV* gene (NM_000243), responsible for the disease was studied by Sanger sequencing. Analysis of the exons and the flanking intronic regions did not reveal the presence of any pathogenic variant. However, this study does not preclude large deletions. Recently, she had an outbreak of the disease with TG levels reaching 54.33 mmol/l and they were rapidly reduced to 4.29 mmol/l following a very strict low-fat diet.

Her brother (III.5), aged 7 years at the time of recruitment, had TG levels fluctuating between 33.22 mmol/l for the highest value and 3.175 mmol/l for the lowest. At recruitment, he presented high TG levels (4.99 mmol/l) and low LDL-C and HDL-C levels (0.51 and 0.36 mmol/l respectively). At the age of 10 years, he was also underweight with a BMI of 14.6 kg/m^2^. Clinically, he presented cutaneous xanthomatosis on his elbows and asthenia. He was diagnosed with splenomegaly consecutive to echography. He presented hemoglobin levels that fluctuated between normal and low. The normal results of hemoglobin electrophoresis allowed to rule out thalassemia. Recently, he presented moderately high levels of TG.

Curiously, TG levels fluctuated concomitantly between the two children and were especially high following the migration from a country to another. For example, the TG levels were the highest upon the arrival of the family to Lebanon, and then they were gradually reduced.

Both children were under fenofibrate but in a discontinuous way, omega 3, vitamin D, folic acid, iron, and a low-fat diet.

Two of their siblings (III.1 and III.4) presented normal TG and TC levels (Table 1). Their parents (II.3 and II.4) presented moderate hypertriglyceridemia according to the 2018 ACC/AHA classification (Table 1) (Grundy et al., 2019). The father aged 45 years was obese.

**TABLE 1:** Lipid measurements and characteristics of the recruited members.

Subject	Age	Gender	BMI at recruitment	TC	TG	LDL-C	HDL-C	Non-HDL-C	ApoB	Plasma PCSK9 levels	p.(Val227Phe) variant in the *LPL* gene	Leucine insertion in the *PCSK9* gene
			*(kg/m* ^*2*^ *)*			*(mmol/l)*			*(g/l)*	*(ng/ml)*		
II.3	45	M	33.4	4.42	3.40	2.07	0.67	3.75	1.16	78.5	+/–	L9/L11
II.4	43	F	25.4	4.92	2.57	2.69	0.93	3.99	1.28	62.8	+/–	L9/L10
III.1	17	M	24.5	3.00	1.53	1.57	0.62	2.38	0.83	62.1	+/–	L10/L11
III.4	9	F	14.8	4.32	0.80	2.69	0.93	3.39	1.13	46.4	+/–	L9/L9
III.5	7	M	13.9	5.46	4.99*	0.51	0.36	5.10	0.41	26.7	+/+	L10/L11
III.6^¥^	6	F	15.7	5.54	7.51*	0.38	0.33	5.21	0.55	35.3	+/+	L9/L10

^¥^sign indicates the proband.

*sign indicates TG value while the patient was under a very strict low-fat diet.

The +/+ sign indicates that the individual is homozygous for the p.(Val227Phe) variant in the *LPL* gene and the +/– sign indicates that the individual is heterozygous for the variant. The L10 designates the p.Leu21dup or p.L15_L16insL in the *PCSK9* gene and the L11 variant designates the p.Leu21tri or p.L15_L16ins2L.

Age at recruitment is given in years. BMI, is given in kg/m^2^. TC, TG, LDL-C, HDL-C, and non-HDL-C levels are given in mmol/l, ApoB levels are given in g/l and PCSK9 levels are given in ng/ml.

ApoB, apolipoprotein B; F, female; HDL-C, high-density lipoprotein cholesterol; LDL-C, low-density lipoprotein cholesterol; LPL, lipoprotein lipase; M, male; PCSK9, proprotein convertase subtilisin/kexin type 9; Non-HDL-C, non high-density lipoprotein cholesterol; TC, total cholesterol; TG, triglycerides.

### PCSK9 measurements

**FIGURE 2:**
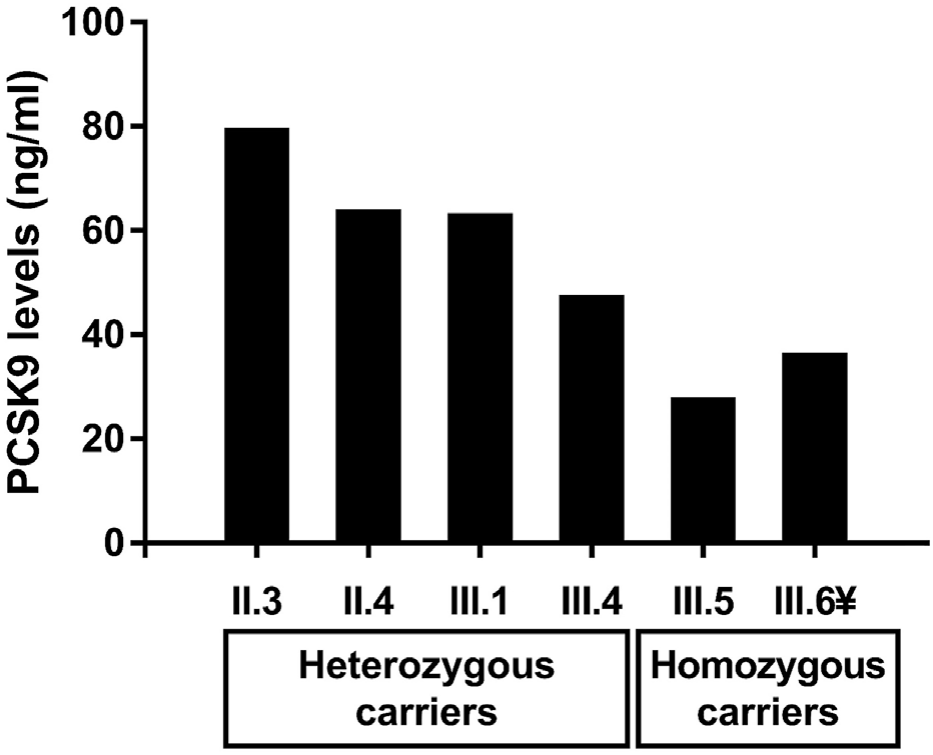
Levels of circulating PCSK9 in heterozygous and homozygous carriers of the p.(Val227Phe) variant in the *LPL* gene in the recruited family. The ¥ sign indicates the proband.

Patients with T1HLP generally present low LDL-C levels and HDL-C levels besides high TG levels (Fojo and Brewer, 1992; Hegele, 2013; Blom et al., 2018; O’Dea et al., 2019). Moreover, many studies have reported a potential correlation between plasma PCSK9 levels and LDL-C, but also triglycerides-rich lipoproteins and investigations linking these actors are being conducted (Lakoski et al., 2009; Druce et al., 2015; Norata et al., 2016; Baragetti et al., 2018; Warden et al., 2020). For these reasons, we measured plasma PCSK9 levels in the recruited members of the family. The results are presented in Table 1 and Figure 2. Homozygous carriers of the p.(Val227Phe) variant (III.5 and III.6) presented a ≈50% decrease in PCSK9 levels compared to heterozygous carriers (II.3, II.4, III.1, and III.4) [median with its interquartile ranges (first quartile–third quartile) of 31.00 (26.70–35.30) ng/ml versus 62.45 (50.33–74.58) ng/ml, respectively] without being significant (*p*-value = 0.13). Moreover, using Spearman correlation PCSK9 levels were positively correlated with age, ApoB levels and with BMI in all family members (*r* = 0.94, *p*-value = 0.017; *r* = 0.89, *p*-value = 0.033 and *r* = 0.94, *p*-value = 0.017, respectively).

### Genetic analysis

We sequenced all the exons and the flanking exon-intron boundaries of the *LPL* gene knowing that most cases of T1HLP are caused by loss-of-function variations in it. Sequencing of this gene revealed the presence of the c.679G > T variation in exon 5 (NM_000237.3) at the homozygous state in both affected children (III.5 and III.6) (Figure 1). This nucleotide change causes a valine to phenylalanine substitution at position 227 of the amino acid chain [p.(Val227Phe)]. The parents (II.3 and II.4) and the two recruited siblings (III.1 and III.4) were heterozygous for the p.(Val227Phe) variation (Figure 1).

In silico analysis revealed that the amino acid valine at position 227 is well conserved among species. The variation is not present in the gnomAD database and was predicted to be disease-causing on Mutation Taster (Grantham Matrix score of 50), deleterious on PROVEAN (with a score of -4.549), with probably damaging consequences on the functionality of the protein according to Polyphen-2 (score of 0.960, sensitivity: 0.63; specificity: 0.92), and presented a CADD score of 25.8 suggesting that this variant is predicted to be among the top 1% of the most deleterious variants in the human genome.

Interestingly, another *LPL* variant in the same codon (p.Val227Ala) was also reported to be responsible for T1HLP (Maruyama et al., 2004; Caddeo et al., 2018).

We also sequenced the *PCSK9* gene to investigate the presence of an eventual variant that might explain the low levels of PCSK9 observed in some individuals of the family. The sequencing revealed the presence of two types of variations in exon 1 of the *PCSK9* gene: the leucine insertion L10 also designated p.Leu21dup or p.L15_L16insL and the leucine insertion L11 also designated p.Leu21tri or p.L15_L16ins2L (Abifadel et al., 2008). The father (II.3) was heterozygous for the L11 variation while the mother (II.4) and one of the affected children (III.6) were heterozygous for the L10 variation. One child (III.4) carried the normal L9 alleles, and two children (III.1 and III.5) were compound heterozygotes for the L10 and L11 alleles. The results are presented in Figure 1.

## Discussion

The p.(Val227Phe) pathogenic variant has been recently reported by Caddeo et al. (2018) in a University Hospital in Gothenburg (Sweden) at the homozygous state in a proband aged 29 years who presented a mean TG level of 18.7 ± 3.2 mmol/l and recurrent pancreatitis episodes. His brother also carried the pathogenic variant. They have likely migrated from the Middle East to Sweden where the center received migrants from Syria and Iran (Caddeo et al., 2018). Functional studies demonstrated the pathogenicity of the p.(Val227Phe) loss-of-function variant. These studies suggested that this variant affects protein production and secretion, but not its degradation. Results showed that the transfection of the HEK 293T/17 cells with the p.(Val227Phe) variant caused a reduction in protein synthesis by 35%–40% and a decrease by at least 80% in the secretion of the LPL in the media of these cells compared to the wild type LPL. Moreover, LPL enzymatic assay showed that LPL activity was absent in the media of the cells transfected with the variant. It is noteworthy that there was no difference in the intracellular degradation rate between the wild type and the mutant *LPL* transfected cells (Caddeo et al., 2018).

We identified the p.(Val227Phe) variant in the *LPL* gene causing T1HLP in a consanguineous Syrian family who migrated to Lebanon. Indeed, this is the first study to measure PCSK9 levels in T1HLP. In this family, homozygous carriers of the p.(Val227Phe) variant presented lower levels of PCSK9 than heterozygous carriers [median (first quartile–third quartile) of 31.00 (26.70–35.30) ng/ml versus 62.45 (50.33–74.58) ng/ml, respectively]. PCSK9 levels were positively correlated with age, ApoB levels and with BMI in all family members (*p*-values = 0.017, 0.033, and 0.017 respectively).Compared to a cohort of 279 Lebanese children of approximately the same age group (8–11 years) in whom PCSK9 levels were measured using the same method [median (first-third quartile) of 64.30 (46.95–93.38) ng/ml] (Azar et al., 2022), the two homozygous children (III.5 and III.6) presented PCSK9 levels that were approximately between the fifth (30.40 ng/ml) and the 7.5th percentile (33.25 ng/ml) of PCSK9 levels in the group, while their heterozygous sibling (III.4) presented PCSK9 levels (46.40 ng/ml) that were approximately within the 25th percentile (46.95 ng/ml). The sibling (III.1) presented PCSK9 levels of 62.1 ng/ml, which is normal compared to a group of 159 Lebanese adolescents aged 15–18 years [median (first-third quartile) of 60.55 (44.51–93.96) ng/ml] (Azar et al., 2022). The presence of loss-of-function variants in the *PCSK9* gene has been studied by sequencing the whole gene. Interestingly, this is the first report of a family with an *LPL* pathogenic variant also carrying variants in *PCSK9*, more precisely two types of leucine insertion variants in the exon 1 of the *PCSK9* gene (Table 1). These polymorphisms occur in the signal peptide region of the PCSK9 protein and are characterized by the insertion of one or two leucines into a stretch of nine leucines (Chen et al., 2005; Yue et al., 2006; Abifadel et al., 2008). They might lead to a structural change in the signal peptide causing an impairment in its cleavage and processing in the endoplasmic reticulum (Yue et al., 2006; Pisciotta et al., 2012; Benito-Vicente et al., 2022)**.** In a study conducted on 1,745 apparently healthy individuals, plasma PCSK9 levels were significantly lower in individuals carrying a leucine insertion in exon 1 of the *PCSK9* gene (Awan et al., 2013). Moreover, the insertion of two leucines in the signal peptide has been reported in a family with familial combined hyperlipidemia and two patients with FH (Abifadel et al., 2008) and *in vitro* studies have shown that it causes a reduction in the secretion of the mature form of PCSK9 compared to the wild type PCSK9 (Benito-Vicente et al., 2022). However, these variations might not alone explain the very low levels of PCSK9 observed in homozygous carriers of the *LPL* variant in our study.

Moreover, homozygous carriers of the p.(Val227Phe) variant in this family presented low levels of LDL-C and HDL-C. This was also observed in the study conducted by Caddeo et al. (2018), as well as in patients suffering from T1HLP described in the literature (O’Dea et al., 2019; Susheela et al., 2021). The low levels of LDL-C in our studied family cannot be explained by the presence of the leucine insertions in the signal peptide of PCSK9. On one hand, the leucine insertion L10 also designated p.Leu21dup or p.L15_L16insL is a common variation associated with lower levels of LDL-C in populations with normal to low LDL-C levels, but also in patients suffering from FH carrying the same p.(Cys681X) mutation in the LDL receptor (*LDLr*) gene (Abifadel et al., 2009). On the other hand, the leucine insertion L11 also designated p.Leu21tri or p.L15_L16ins2L is a rare variant that has been associated with familial combined hyperlipidemia and was found at a low frequency in subjects presenting LDL-C levels of 2.96–4.90 mmol/L and coronary lesions in the American population (Chen et al., 2005; Abifadel et al., 2008; Benito-Vicente et al., 2022). In fact, low levels of LDL-C and HDL-C in T1HLP may be explained by the disruption in the activity of the LPL (Hegele et al., 2018). Variations in LPL activity in humans result in changes in lipoproteins metabolism (Goldberg et al., 1990). Indeed, low or absent activity of LPL results in an impairment in the conversion of TG-rich particles to their remnant lipoproteins, including chylomicron remnants, VLDL, VLDL remnants, intermediate density lipoprotein and LDL. The subsequent decrease in the available cholesterol from VLDL, LDL, and peripheral tissues causes a decrease in HDL-C levels (Hegele et al., 2018). Other studies attribute the low levels of LDL-C and HDL-C to an increase in their catabolism, besides the decrease in their synthesis (Fojo and Brewer, 1992). Interestingly, the low levels of LDL-C might explain the observed low levels of plasma PCSK9. In fact, many studies have shown a positive correlation between circulating plasma PCSK9 and LDL-C (Shapiro et al., 2019). It is suggested that this correlation is due to the fact that PCSK9 acts as an important regulator of LDL metabolism through targeting the LDLR for lysosomal degradation, but also due to a direct interaction between PCSK9 and LDL (Abifadel et al., 2010; Tavori et al., 2015). Further studies of PCSK9 levels in patients with hyperchylomicronemia would be interesting in order to verify if all homozygous patients with T1HLP have low PCSK9 levels, or if it is specific to the patients in the studied family, and to decipher the causes of this decrease, as well as the mechanism, the role of PCSK9, and its correlation with ApoB levels (Ayoub et al., 2021).

It is noteworthy that anemia has been described as a clinical sign of T1HLP that occurs in some cases (Rahalkar and Hegele, 2008; Brahm and Hegele, 2013). In a case series of infants with T1HLP, 7 out of 16 infants presented normocytic anemia which cause was unknown, and both males and females were affected (Feoli-Fonseca et al., 1998). In another study conducted to determine the phenotype-genotype relationships between different subgroups of T1HLP, all the female participants (*n* = 7) suffered from anemia (Chokshi et al., 2014).

Although the parents (II.3 and II.4) were heterozygous carriers of the p.(Val227Phe) variant, they presented moderately high levels of TG. The phenotypic expression of heterozygous LPL deficiency is not clinically and biochemically well described yet. It has been reported that heterozygous carriers of one defective allele in the *LPL* gene do not present chylomicronemia nor other manifestations of the disease. They may present normal or moderately increased fasting TG levels, especially when fed a high-calorie, high-fat diet (Miesenböck et al., 1993; Hölzl et al., 2000; Abifadel et al., 2004). The presence of precipitating factors such as age, obesity, pregnancy, hyperinsulinemia, and lipid-raising drugs would contribute to the phenotypic expression of heterozygous LPL deficiency (Wilson et al., 1990; Abifadel et al., 2004).

The identification of heterozygous carriers of an *LPL* mutation is of major importance, especially in countries with a high frequency of consanguineous marriages (Abifadel et al., 2004). It is also necessary for the prevention of precipitating factors knowing that the lipoprotein phenotype in heterozygous carriers of a defective *LPL* allele has been considered atherogenic (Reymer et al., 1995; Bijvoet et al., 1996; Wittrup et al., 1997; Hölzl et al., 2000).

The high prevalence of consanguineous marriages in Middle Eastern countries increases the risk of autosomal recessive genetic diseases (Al-Herz and Al-Mousa, 2013) such as hyperchylomicronemia.

A study conducted to determine the prevalence of consanguineous marriages in Syria showed that the overall frequency was 30.3% in urban areas and 39.8% in rural ones, with an overall rate of 35.4%. In some provinces, the frequency could reach 67.5%. Among this type of marriage, first cousins’ marriages were the most common with a rate of 20.9% (Othman and Saadat, 2009). It is noteworthy that the natality rate increases in refugees’ camps or migrant populations, as well as the risk of consanguineous marriages.

To date, more than 200 pathogenic variants in the *LPL* gene have been reported to cause T1HLP (Caddeo et al., 2018). However, rare cases have been described in the Middle East and the Mediterranean regions. A summary of the described mutations in this region is presented in Table 2.

**TABLE 2:** Summary of the described variations in the *LPL* gene in the Middle East and the Mediterranean regions.

Region	Amino acid variation in the *LPL* gene according to the article	Case presentation	References
Lebanon, 2004	Homozygous for the p.(Asp174Val)/p.(Asp201Val) variant (according to the original/present nomenclature)	A 34-year-old male with TG levels of 34.3 mmol/l lowered to 6.37 mmol/l under medication and a low-fat diet.	Abifadel et al. (2004)
		A 7-year-old boy with a TG peak of 30.45 mmol/l during an episode of pancreatitis and recurrent abdominal pain since 3 years old	
Greece, 2004	Compound heterozygous for the p.(Gly188Glu) and p.(Met301Arg) variants (according to the original nomenclature)	A 32-day-old girl with TG levels of 169.3 mmol/l at the time of admission to the hospital and rapidly lowered to 11.2 mmol/l 10 days after the administration of medium-chain triglycerides enriched milk	Kavazarakis et al. (2004)
Middle East, 2013	Homozygous for the p.(Arg270His) variant (according to the present nomenclature)	A 2-month-old Arab infant with a TG peak of 276.6 mmol/l rapidly lowered to 4.93 mmol/l at the time of discharge from the hospital after the administration of a medium-chain triglycerides-rich diet.	Hegele et al. (2018)
Morocco, 2015	Homozygous for the p.(Ser286Arg) variant (according to the present nomenclature)	A 19-year-old girl with TG levels of 199 mmol/l lowered to 14.15 mmol/l 2 months after the administration of an appropriate diet, a maximal dose of fenofibrate and simvastatin along with heparin and insulin	Bouabdellah et al. (2015)
Middle East, 2016	(p.Gly256Thrfs*26) (according to the present nomenclature)	A 19-year-old female with TG levels of 60.7 ± 7 mmol/l and a 32-year-old male with TG levels of 31.7 ± 7.8 mmol/l	Pingitore et al. (2016)
	(p.Met404Arg) (according to the present nomenclature)	A 28-year-old male with TG levels of 45 ± 26.9 mmol/l	
Some of them from the Middle East, 2017	Compound heterozygous for the p.(Trp113Arg), the p.(Gly215Glu), and the p.(Met404Arg) variants (according to the present nomenclature)	A 49-year-old female with TG levels of 39.7 ± 13.6 mmol/l	Caddeo et al. (2018)
	Heterozygous for the p.(Ser220Arg) variant (according to the present nomenclature)	A 69-year-old male with TG levels of 19.6 ± 9.4 mmol/l	Caddeo et al. (2018)
	Homozygous for the p.(Val227Phe) variant (according to the present nomenclature)	A 29-year-old male with TG levels of 18.7 ± 3.2 mmol/l	Caddeo et al. (2018)

The original nomenclature considers the amino acid numbering of the mature protein [without the signal peptide of 27 amino acids (Deeb and Peng, 1989)], while the present international nomenclature considers the initiator methionine as the first amino acid of the LPL (NP_000228.1).

## Conclusion

This is the first study to measure plasma PCSK9 levels in T1HLP. We found that homozygous affected patients presented low levels of PCSK9 compared to heterozygous non-affected members of the family and to children from the same age group. Further studies of PCSK9 levels in patients with hyperchylomicronemia would be interesting in order to verify if all homozygous patients with T1HLP have low PCSK9 levels. This would help decipher the causes of this decrease, as well as the mechanism and the role of PCSK9 in this disease. It might also be interesting to elucidate pathways linking PCSK9 to apolipoprotein B or eventually to triglycerides-rich lipoproteins metabolism.

The identification of the same mutation in the *LPL* gene in two distinct families with T1HLP originated most probably from the same region in Syria and that migrated to either Lebanon or Sweden, should lead us to search for this mutation as the first cause of hyperchylomicronemia in patients originated from Syria. A founder effect could be hypothesized but needs further investigation and cases to be confirmed.

Public awareness and education concerning the medical risks of consanguineous marriages are important and should be included in the international effort and politics that provide care, birth control, and genetic counseling when needed. They might help in decreasing the emergence of inherited autosomal recessive diseases.

Finally, handling a metabolic disease that needs specific care and compliance to a drastic regimen or treatment to prevent some fatal complications is not easy, especially in a crisis. The main solution is to prevent the emergence of these diseases by raising awareness of the risks of consanguineous marriages.

It is more and more urgent to address genetic lipid disorders and more generally genetic diseases that need special treatment, diagnosis, management, and prevention especially in refugee populations or in war or economic crisis countries.

## Data Availability

The datasets for this article are not publicly available due to concerns regarding participant/patient anonymity. Requests to access the datasets should be directed to the corresponding author.
